# Delayed cerebral vasculopathy following pneumococcal meningitis: a case report

**DOI:** 10.1093/omcr/omaf051

**Published:** 2025-05-28

**Authors:** Mohammad Aladawi, Mohamed Elfil, Pashayar Lookian, Zaid Najdawi, Mahmoud A Fayed, Edson DeOliveira, Steven Phillips

**Affiliations:** Department of Neurology, University of Alabama Medical Center, 1813 6th Avenue South, RWUH M226, Birmingham, AL 35294, United States; Department of Neurology, University of Miami/Jackson Health System, 1501 NW 9th Ave, Miami, FL 33101, United States; Department of Neurosurgery, University of Nebraska Medical Center, 139 S 40th St, Omaha, NE 68131, United States; Department of Neurological Sciences, University of Nebraska Medical Center, 619 S 42nd St, Omaha, NE 68198, United States; Department of Internal Medicine, Mansoura University Hospital, 60 El Gomhoureya Street, Mansoura 35516, Egypt; Department of Neurology, Indiana University School of Medicine, 355 W. 16th Street Goodman Hall Suite 4700, Indianapolis, IN 46202, United States; Department of Neurological Sciences, University of Nebraska Medical Center, 619 S 42nd St, Omaha, NE 68198, United States

**Keywords:** acute bacterial meningitis, delayed cerebral vasculopathy, ischemic stroke, vasospasm, Milrinone

## Abstract

Acute bacterial meningitis (ABM) remains a common disease, especially in developing countries. Although morbidity and mortality have improved with advances in medicine, significant neurologic complications of meningitis still occur. Delayed cerebral vasculopathy (DCV) is a unique complication following ABM leading to ischemic strokes and poor functional outcomes. We describe a case of a 66-year-old man with repeated acute infarcts from DCV due to ABM, notably in the absence of empiric dexamethasone treatment, which was not administered as meningitis was diagnosed post-antibiotic initiation. New areas of acute ischemia were noted on repeat imaging on days 6, 24, and 30 of admission. Vascular imaging revealed multifocal vascular stenosis, consistent with vasospasm that was successfully treated with Milrinone.

## Introduction

Pneumococcal meningitis is a severe neuro-infectious disease associated with increased rates of mortality and long-term neurological sequelae in survivors [1]. Among the neurological complications of pneumococcal meningitis is delayed cerebral vasculopathy (DCV), a rare but serious complication of acute bacterial meningitis (ABM), occurring in approximately 4% of cases, with some smaller retrospective studies reporting an incidence as high as 10% [2, 3]. DCV is often associated with poor outcomes, including high morbidity and mortality due to recurrent strokes and progressive neurological decline [4].

While previous reports have highlighted the challenges of managing DCV, this case provides novel insights into both the limitations of initiating corticosteroid therapy and the use of milrinone as a potential therapeutic intervention. We describe the complex course of a 66-year-old male patient diagnosed with pneumococcal meningitis, complicated by DCV, which led to multiple cerebral infarcts and vasospasm that improved with milrinone administration. By sharing this intricate case, we strive to shed light on the challenging aspects of managing pneumococcal meningitis complicated by DCV and enhance the existing body of clinical knowledge.

## Case report

**Figure 1:**
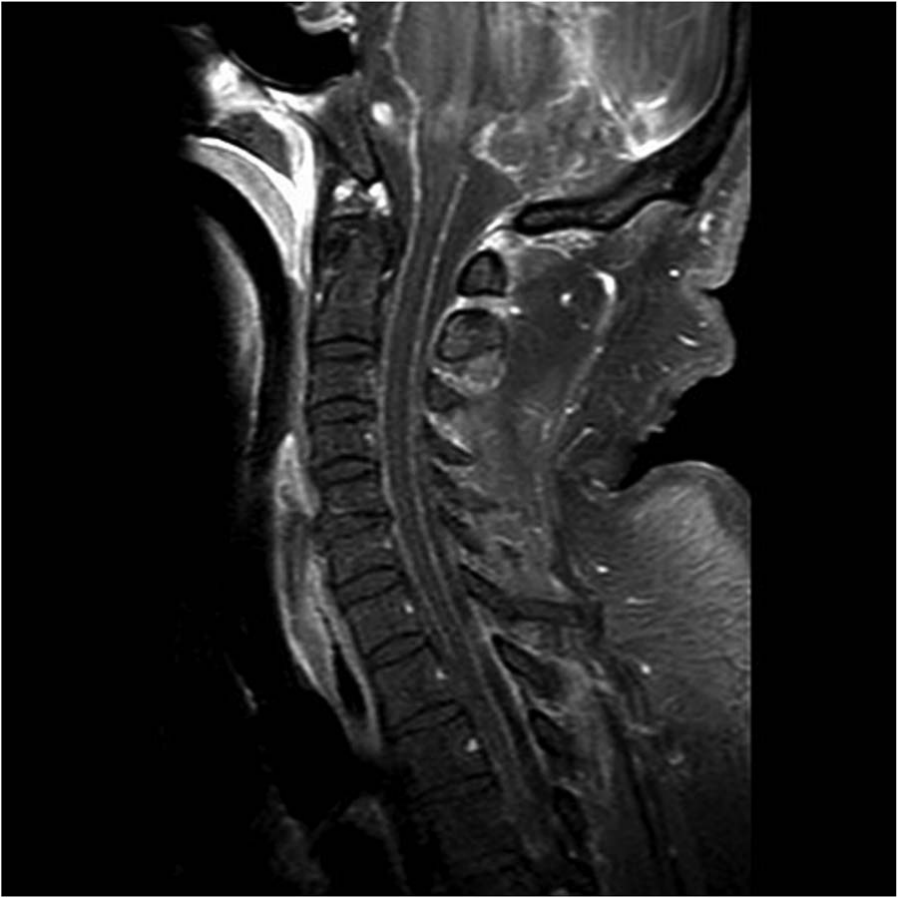
MRI of the spinal cord revealing leptomeningeal enhancement along the cervical and thoracic spinal cord and cauda equina nerve roots.

**Figure 2:**
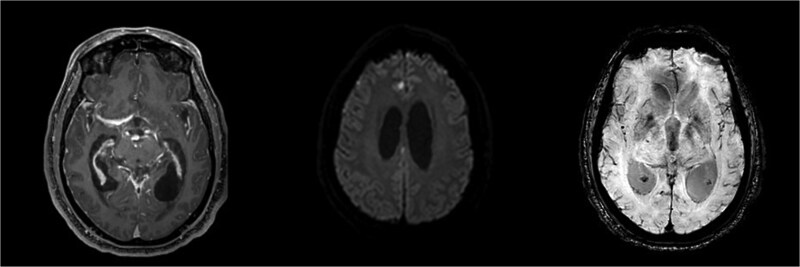
MRI of the brain revealing ventriculitis, hydrocephalus, microhemorrhages, and small punctate infarcts in the inferior frontal lobes and right paramedian frontal lobe.

A 66-year-old male presented with a two-week history of acutely worsening back pain and two days of altered mental status. He was intubated for airway protection. Initial exam was limited due to paralytics but it showed equally reactive pupils and withdrawal to pain in all extremities. Labs showed white blood cell (WBC) count of 12.1 × 10^9^/L, erythrocyte sedimentation rate (ESR) of 128 mm/h, C-reactive protein (CRP) of 53.6 mg/L, and procalcitonin of 26.20 ng/mL. Empiric vancomycin and cefepime were started. Computed tomography (CT) scan of the head revealed moderate ventricular prominence. CT of the chest, abdomen, and pelvis were unremarkable aside from mild atelectasis. Magnetic resonance imaging (MRI) scan of the spinal cord revealed diffuse leptomeningeal enhancement along the cervical and thoracic spine with L4-L5 facet joint effusions and small paraspinal muscle abscesses (Fig. 1). Brain MRI showed leptomeningeal enhancement, communicating hydrocephalus, ventriculitis, and punctate infarcts in the inferior frontal lobes and right paramedian frontal lobe (Fig. 2).

Blood cultures were positive for *Streptococcus pneumoniae*. Cerebrospinal fluid (CSF) analysis showed WBC 113 cells/μL, protein 319 mg/dL, and glucose < 10 mg/dL, with CSF cultures also positive for *S. pneumoniae*. Accordingly, antibiotics were deescalated to ceftriaxone. Dexamethasone was not given as meningitis was diagnosed post-antibiotic initiation.

**Figure 3:**
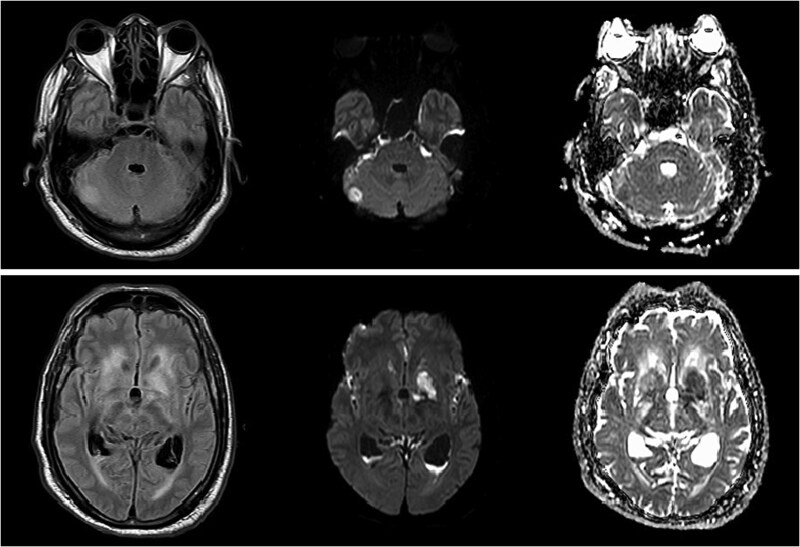
Follow-up brain MRI revealing acute infarcts in the right cerebellum and left basal ganglia.

The hospital course was complicated by worsening neurologic status. On day 6, the patient had an asymmetric pupil with an MRI brain confirming acute infarcts in the right cerebellum and left basal ganglia (Fig. 3). Transthoracic and Transesophageal Echocardiogram was negative for endocarditis.

**Figure 4:**
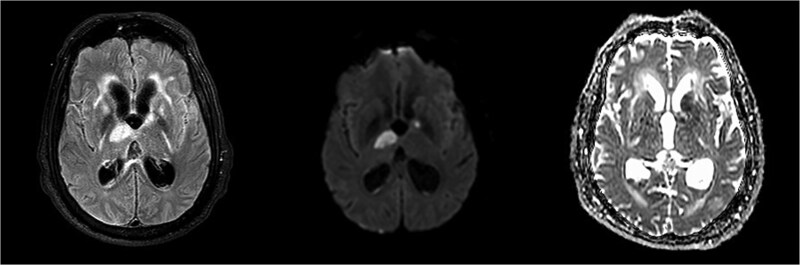
Repeat brain MRI showing additional acute infarcts in the right thalamus and left basal ganglia.

**Figure 5:**
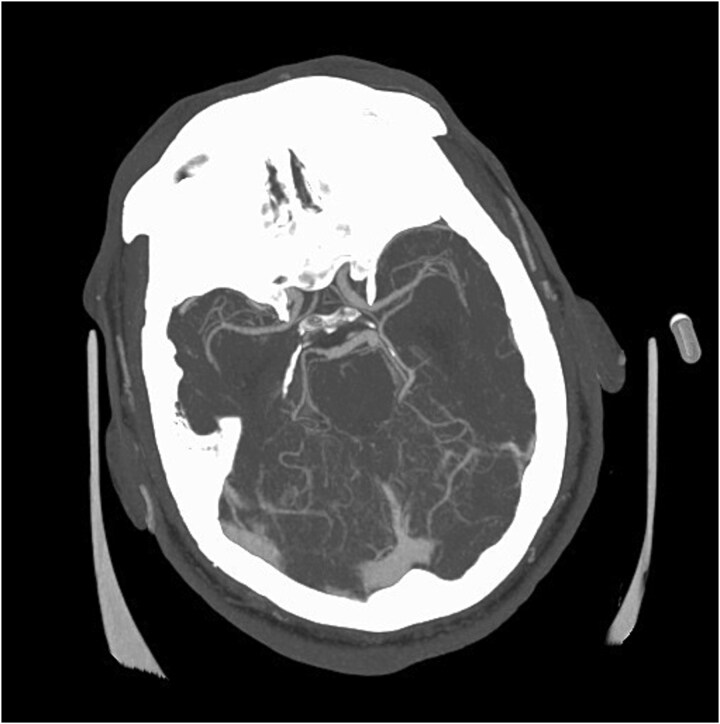
Repeat CTA head demonstrating new multifocal intracranial vessels stenosis.

On day 24, the patient’s developed left hemiplegia. Repeat brain MRI showed additional acute infarcts in the right thalamus and left basal ganglia (Fig. 4). Computed tomography angiography (CTA) of the head and neck were unremarkable. Repeat CSF and blood cultures were negative. On day 30, repeat CTA showed multifocal intracranial stenosis and a new left cerebellar hypoattenuation (Fig. 5).

**Figure 6:**
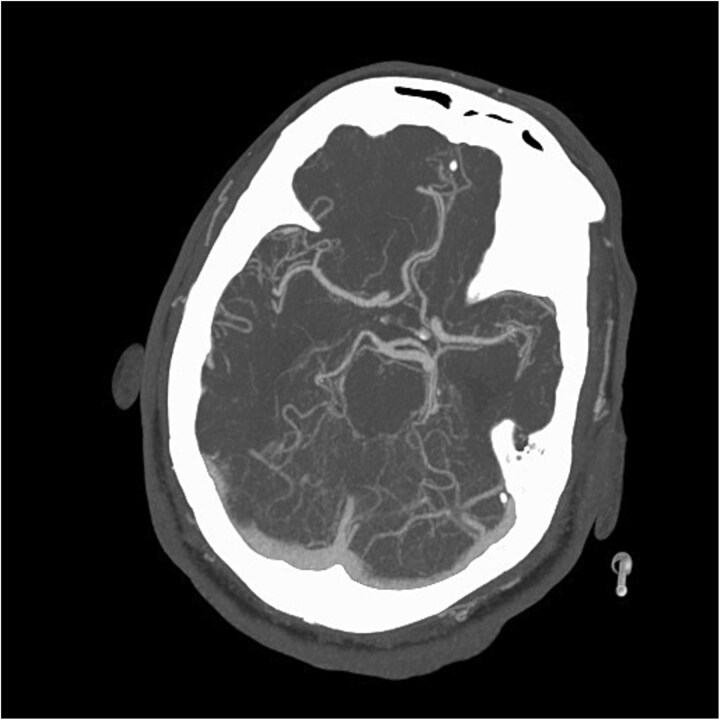
Repeat CTA head showing improved caliber of the anterior and posterior cerebral arteries following Milrinone treatment.

The patient was started on a trial of milrinone, which led to improved anterior and posterior cerebral artery caliber over two days on repeat CTA, and the patient was transitioned to cilostazol 100 mg twice daily (Fig. 6). However, he remained comatose, and comfort care was initiated on day 35. He subsequently passed away.

## Discussion

This case highlights the impact of delayed administration of empiric steroids in ABM and its potential contribution to the development of DCV [5]. In this patient, dexamethasone was not administered as bacterial meningitis was diagnosed only after the initiation of antibiotics. Several factors contributed to the delay in diagnosis, including the atypical presentation and the initial focus on alternative causes of illness. This underscores the necessity of maintaining a high index of suspicion for bacterial meningitis, particularly in critically ill patients with systemic signs of infection. Current guidelines recommend administering corticosteroids before or at the same time as the first dose of antibiotics in suspected cases of pneumococcal meningitis to mitigate inflammatory-mediated complications [6, 7].

Early administration of dexamethasone has been shown to attenuate the intense inflammatory response seen in ABM, which may contribute to blood–brain barrier dysfunction and subsequent vascular complications [8]. The absence of early steroid treatment in this case may have amplified the inflammatory response, increasing the risk of vascular endothelial damage and predisposing the patient to progressive cerebrovascular complications [9].

DCV is a complex phenomenon involving para-infectious vasculitis, post-infectious autoimmune-mediated vasculopathy, cerebral vasospasm, and thrombosis [4, 10, 11]. While molecular pathways involving cytokine release, complement activation, and endothelial dysfunction play a role in these processes, the primary clinical challenge remains timely recognition and intervention [12]. Given the limited understanding of the precise molecular mechanisms underlying DCV, future studies should explore targeted therapies aimed at mitigating inflammatory damage, such as matrix metalloproteinase inhibitors and complement pathway modulators [13, 14].

In managing DCV, treatment strategies have largely been extrapolated from approaches used in subarachnoid hemorrhage-associated vasospasm [15, 16]. Vasodilatory agents, including milrinone, have shown promise in improving vascular caliber and preventing further ischemic injury [17]. This case demonstrated a transient improvement in vessel caliber following milrinone administration, although it did not translate to a favorable clinical outcome. The role of immunomodulatory therapies, including high-dose corticosteroids in the later stages of DCV, warrants further investigation. Some reports suggest that pulse-dosing steroids may be beneficial in cases where vasculitis is the predominant mechanism of DCV [9, 18].

Ultimately, this case highlights the critical importance of early administration of corticosteroids in suspected bacterial meningitis. It also emphasizes the need for ongoing research into the optimal management of DCV, including identifying patients at the highest risk for cerebrovascular complications and refining treatment approaches that address both inflammation and vascular dysfunction.

## References

[1] Castelblanco RL, Lee M, Hasbun R. Epidemiology of bacterial meningitis in the USA from 1997 to 2010: a population-based observational study. Lancet Infect Dis 2014;14:813–9. 10.1016/S1473-3099(14)70805-9 25104307

[2] Boix-Palop L, Fernandez T, Pelegrin I. et al. Delayed cerebral vasculopathy in pneumococcal meningitis: epidemiology and clinical outcome. A cohort study. Int J Infect Dis 2020;97:283–9. 10.1016/j.ijid.2020.06.005 32531430

[3] Gallegos C, Tobolowsky F, Nigo M. et al. Delayed cerebral injury in adults with bacterial meningitis: a novel complication of adjunctive steroids? Crit Care Med 2018;46:e811–4. 10.1097/CCM.0000000000003220 29746358

[4] Depoortere S, Toeback J, Lunskens S. et al. Delayed cerebral thrombosis complicating bacterial meningitis. Acta Clin Belg 2022;77:462–9. 10.1080/17843286.2021.1873583 33455561

[5] Brouwer MC, McIntyre P, Prasad K. et al. Corticosteroids for acute bacterial meningitis. Cochrane Database Syst Rev 2015;2018:CD004405. 10.1002/14651858.CD004405.pub5 PMC6491272 26362566

[6] de Gans J, van de Beek D. European dexamethasone in adulthood bacterial meningitis study I. Dexamethasone in adults with bacterial meningitis. N Engl J Med 2002;347:1549–56. 10.1056/NEJMoa021334 12432041

[7] Tunkel AR, Hartman BJ, Kaplan SL. et al. Practice guidelines for the management of bacterial meningitis. Clin Infect Dis 2004;39:1267–84. 10.1086/425368 15494903

[8] Pugin D, Copin JC, Goodyear MC. et al. Persisting vasculitis after pneumococcal meningitis. Neurocrit Care 2006;4:237–40. 10.1385/NCC:4:3:237 16757830

[9] Chan OW, Lin JJ, Hsia SH. et al. Methylprednisolone pulse therapy as an adjuvant treatment of Streptococcus pneumoniae meningitis complicated by cerebral infarction-a case report and review of the literature. Childs Nerv Syst 2020;36:229–33. 10.1007/s00381-019-04485-6 31897636

[10] Schut ES, Brouwer MC, de Gans J. et al. Delayed cerebral thrombosis after initial good recovery from pneumococcal meningitis. Neurology 2009;73:1988–95. 10.1212/WNL.0b013e3181c55d2e 19890068

[11] Steiner I . Past as prologue: delayed stroke as a parainfectious process of bacterial meningitis? Neurology 2009;73:1945–6. 10.1212/WNL.0b013e3181c55d43 19890067

[12] Coutinho LG, Grandgirard D, Leib SL. et al. Cerebrospinal-fluid cytokine and chemokine profile in patients with pneumococcal and meningococcal meningitis. BMC Infect Dis 2013;13:326. 10.1186/1471-2334-13-326 23865742 PMC3717124

[13] Koelman DLH, Brouwer MC, van de Beek D. Targeting the complement system in bacterial meningitis. Brain 2019;142:3325–37. 10.1093/brain/awz222 31373605 PMC6821383

[14] Leib SL, Leppert D, Clements J. et al. Matrix metalloproteinases contribute to brain damage in experimental pneumococcal meningitis. Infect Immun 2000;68:615–20. 10.1128/IAI.68.2.615-620.2000 10639424 PMC97183

[15] Norman S, Rosenberg J, Sundararajan SH. et al. Management of refractory bacterial meningitis-associated cerebral vasospasm: illustrative case. J Neurosurg Case Lessons 2023;5. 10.3171/CASE22418 PMC10550601 36794739

[16] Taqui A, Koffman L, Hui F. et al. Intra-arterial vasodilator therapy for parainfectious cerebral vasospasm. J Neurol Sci 2014;340:225–9. 10.1016/j.jns.2014.02.028 24655738

[17] Lakhal K, Hivert A, Alexandre PL. et al. Intravenous Milrinone for cerebral vasospasm in subarachnoid Hemorrhage: the MILRISPASM controlled before-after study. Neurocrit Care 2021;35:669–79. 10.1007/s12028-021-01331-z 34478028

[18] Czartoski T, Hallam D, Lacy JM. et al. Postinfectious vasculopathy with evolution to moyamoya syndrome. J Neurol Neurosurg Psychiatry 2005;76:256–9. 10.1136/jnnp.2004.041046 15654044 PMC1739483

